# Importance of Circulating Leptin and Adiponectin in the Causal Pathways Between Obesity and the Development of Colorectal Cancer in Japanese Men

**DOI:** 10.2188/jea.JE20230148

**Published:** 2024-12-05

**Authors:** Masataka Taguri, Aya Kuchiba, Taiki Yamaji, Norie Sawada, Atsushi Goto, Motoki Iwasaki, Shoichiro Tsugane

**Affiliations:** 1 Department of Health Data Science, Tokyo Medical University, Tokyo, Japan; 2 Division of Biostatistical Research, Institution for Cancer Control/Biostatistics Division, Center for Research Administration and Support, National Cancer Center, Tokyo, Japan; 3 Division of Epidemiology, National Cancer Center Institute for Cancer Control, Tokyo, Japan; 4 Division of Cohort Research, National Cancer Center Institute for Cancer Control, Tokyo, Japan; 5 National Institute of Health and Nutrition, National Institutes of Biomedical Innovation, Health and Nutrition, Tokyo, Japan; 6 Graduate School of Health Innovation, Kanagawa University of Human Services, Kawasaki, Japan; 7 Department of Health Data Science, Graduate School of Data Science, Yokohama City University, Yokohama, Japan

**Keywords:** causal mediation analysis, obesity, colorectal cancer, adipokines, multiple mediators, prospective cohort study

## Abstract

**Background:**

The mechanistic associations between obesity and risk of colorectal cancer (CRC) remain unclear. Here, using body mass index (BMI) as an obesity indicator, we decomposed the total effects of obesity on the risk of CRC into: (1) direct effects, which are possibly mediated by unmeasured or currently unknown factors; (2) indirect effects mediated by circulating leptin and adiponectin; and (3) indirect effects that are not mediated by circulating leptin and adiponectin but by hyperinsulinemia and chronic inflammation (assessed via circulating connecting peptide and C-reactive protein, respectively).

**Methods:**

We adopted a causal mediation framework, using data from a large prospective cohort study of 44,271 Japanese men.

**Results:**

BMI was not associated with the risk of CRC due to direct and indirect effects that were not mediated by circulating leptin and adiponectin. By contrast, individuals with BMIs of 25.0–27.4 kg/m^2^ (risk ratio 1.29; 95% confidence interval, 0.98–1.69) and ≥27.5 kg/m^2^ (risk ratio 1.28; 95% confidence interval, 0.98–1.68) had a higher risk of CRC due to indirect effects of circulating leptin and adiponectin.

**Conclusion:**

Our mediation analyses suggest that the association between BMI and CRC risk may be largely mediated by a pathway involving circulating leptin and adiponectin.

## INTRODUCTION

**Figure 1.**
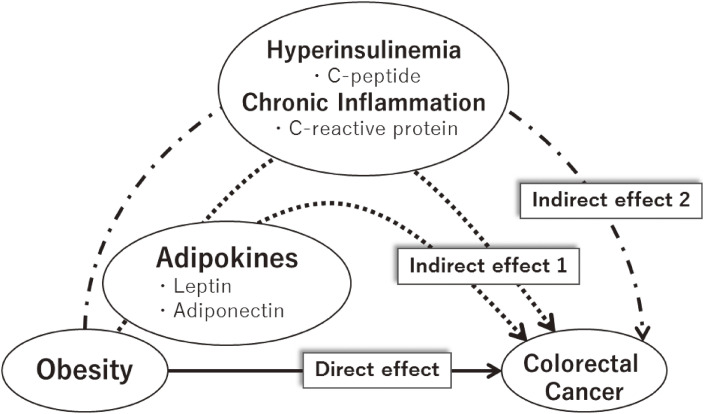
Causally directed acyclic graph illustrating the direct and indirect causal effects between obesity and colorectal cancer. Direct effect: direct effects of obesity, which are possibly mediated by unmeasured or currently unknown factors. Indirect effect 1: indirect effects of obesity that are mediated by circulating leptin and adiponectin. Indirect effect 2: indirect effects of obesity that are not mediated by circulating leptin and adiponectin but by hyperinsulinemia and chronic inflammation (represented by circulating C-peptide and C-reactive protein, respectively). The presence of arrows in Figure 1 indicates the existence of any of six effects described in Statistical analysis. However, the individual representation of each of six effects in this figure is not feasible. Please also refer to the explanations in eTable 1.

Obesity is a well-known risk factor for the development of colorectal cancer (CRC).^1^ Accumulating evidence suggests that adipose tissue, long considered an inert energy storage deposit, functions as an active endocrine organ, releasing a wide range of biologically functional molecules collectively known as adipokines.^2^ The adipokines leptin and adiponectin are predominantly secreted by adipocytes and have the potential to modulate the degree of hyperinsulinemia and chronic inflammation,^2^ both of which are closely associated with colorectal carcinogenesis.^3^ Hyperinsulinemia and chronic inflammation are not completely controlled by leptin and adiponectin, and their effects on the development of CRC cannot be fully explained by the regulation of hyperinsulinemia and chronic inflammation.^4^^,^^5^ Furthermore, it is possible that currently unknown factors mediate the association between obesity and CRC. The complex mechanistic pathways are illustrated in Figure 1.

We used a causal mediation framework to describe the relative importance of mechanistic pathways mediating the association between obesity and CRC development. Causal mediation analysis was initially introduced in social science research.^6^ However, traditional mediation analysis has important methodological limitations concerning its applicability to models with interactions or nonlinearities. The causal inference literature further addresses mediation problems by employing the notion of counterfactuals, which address interactions or nonlinearities and provide causal interpretations and assumptions for identification.^7^^,^^8^ Even though the majority of the methodological literature has focused on mediation analyses with a single mediator,^9^^–^^11^ recent advances in causal mediation frameworks have enabled mediation analysis with multiple mediators.^12^^–^^15^

In this study, we decomposed the total effects of obesity on the 10-year incidence of CRC into three pathways on the basis of new approaches developed by VanderWeele et al^16^ and Daniel et al^12^: (1) direct effects, which are possibly mediated by unassessed or currently unknown factors; (2) indirect effects that are mediated by circulating leptin and adiponectin; and (3) indirect effects that are not mediated by circulating leptin and adiponectin but by hyperinsulinemia and chronic inflammation, assessed via circulating connecting peptides (C-peptides) and C-reactive proteins (CRPs), respectively (Figure 1). We then assessed the relative importance of the three pathways that connect obesity to the risk of CRC. Importantly, four biomarkers that represent the indirect effects of obesity have been associated with an increased risk of CRC.^17^^–^^19^

## METHODS

### Study population

The study cohort was part of the Japan Public Health Center (JPHC)-based prospective study, which was launched in 1990 and 1993 for Cohorts I and II, respectively. The study design has been described in detail previously.^20^ Cohorts I and II consisted of all registered Japanese residents aged 40–59 years from five public health center (PHC) areas and those aged 40–69 years from six PHC areas across Japan, respectively. In the current analysis, one PHC area was excluded because incidence data were not available. Additionally, we restricted the study participants to men because we previously noted a positive association between obesity and CRC in men alone.^21^ Finally, we defined a population-based cohort of 48,376 men. This study was approved by the Institutional Review Board of the National Cancer Center, Tokyo, Japan (approval no. 2001-021 and 2003-055).

### Weight, height, and other variables

At baseline, participants completed a self-administered questionnaire that assessed demographic characteristics, personal medical history, family history of cancer, smoking, alcohol consumption, dietary habits, physical activity, and other lifestyle factors. The respondents also reported their current height (cm) and weight (kg). The body mass index (BMI) was calculated as weight (kg) divided by height (m^2^). Self-reported height and weight data were validated in our previous study.^22^ We classified BMI into three categories based on the World Health Organization classification for Asians (<25.0, 25.0–27.4, and ≥27.5 kg/m^2^).^23^

### Cohort follow-up and identification of CRC cases

The participants were followed up until December 31, 2009. We determined the incidence of CRC based on hospital records and population-based cancer registries in the study area. CRC cases were coded as C18.0–C20.9, according to the International Classification of Diseases for Oncology.^24^ We calculated the person-years of follow-up for each participant from the completion of the baseline questionnaire to the date of CRC diagnosis, date of emigration from the study area, date of death, or end of follow-up (December 31, 2009), whichever occurred first. Over a follow-up period of 738,114 person-years, we identified 1,389 new CRC cases.

### Mediators: leptin, adiponectin, C-peptide, and CRP

In our previous studies on biomarkers and CRC, we conducted a nested case-control study.^25^^–^^29^ Up to December 31, 2003, 196 out of 14,004 men who had returned a baseline questionnaire, reported no cancer diagnosis, and provided blood samples were identified as having CRC. Using incidence-density sampling, two controls were chosen from among participants who had no prior history of CRC.

Biomarker data were available only for participants in the nested case-control study (196 cases and 392 controls). Multiple imputations (MIs) were used for missing biomarker data from the full cohort dataset.^30^ We used a regression model to impute the natural logarithm of plasma concentrations of leptin, adiponectin, C-peptide, and CRP. Each imputation model included BMI, age, PHC area, history of diabetes, pack-years of smoking, alcohol consumption, leisure-time physical activity, family history of cancer, CRC status, baseline cumulative hazard at the time of observation, and the last three biomarkers. 

### Statistical analysis

We adjusted for the following potential confounders in accordance with our previous studies on leptin, adiponectin, C-peptide, and CRP^25^^,^^27^^,^^29^: age (continuous), area (10 PHC areas), pack-years of smoking (non-smoker, <20, between ≥20 and <40, and ≥40 pack-years), alcohol consumption (non-drinker, occasional drinker; and <150, between ≥150 and <300, between ≥300 and <450, and ≥450 g ethanol/week), leisure time of physical activity (rarely, 1–3 days/month, 1–2 days/week, 3–4 days/week, and almost every day), and family history of cancer (yes or no). Among the 48,376 men, we analyzed complete data (no missing potential confounding factors) from 44,271 men.

For individual *i*, let *A* denote BMI (categorized as <25.0, 25.0–27.4, ≥27.5 kg/m^2^); let *Y* denote the 10-year incidence of CRC (1: occurred, 0: did not occur); let **L** = (*L*_1_, *L*_2_) denote adipocyte-derived biomarkers, adiponectin (*L*_1_) and leptin (*L*_2_), and let **M** = (*M*_1_, *M*_2_) denote the other biomarkers of interest, C-peptide (*M*_1_) and CRP (*M*_2_); and let **C** denote a set of confounding factors that may affect the exposure, mediator, and outcome (Figure 1). Owing to positively skewed distributions, all four mediators were log-transformed before the mediation analysis.

We used a potential outcome framework to conduct a causal mediation analysis.^31^^,^^32^ Let *Y_a_*, **L***_a_*, and **M***_a_* denote the potential outcome and mediators, respectively, which would be observed if *A* were set to *a*. Likewise, let *Y_a_***_lm_** denote the potential outcome that would be observed if *A* were set to *a*, **L** were set to **l**, and **M** were set to **m**. We also used consistency and composition assumptions^10^ (See eMaterial 1 for further details).

Even if Figure 1 is interpreted as a non-parametric structural equation model with independent errors,^8^ we cannot identify the effect mediated by both **L** and **M** (*A*→**L**→**M**→*Y*) and the effect mediated by **L** but not **M** (*A*→**L**→*Y*).^33^ However, we can identify the effects through (1) a direct pathway (*A*→*Y*), (2) pathways involving **L** (the combination of *A*→**L**→**M**→*Y* and *A*→**L**→*Y*), and (3) pathways involving **M** but not **L** (*A*→**M**→*Y*), as shown in Figure 1, and the non-parametric structural equation model with independent errors.^16^^,^^33^ Furthermore, we did not need to assume the direction of the causal effect between adiponectin (*L*_1_) and leptin (*L*_2_), and C-peptide (*M*_1_) and CRP (*M*_2_), because we treated (*L*_1_, *L*_2_) and (*M*_1_, *M*_2_) as joint mediators.^13^^,^^15^

Using potential outcomes, we obtained the following decomposition of the total effect on the risk ratio scale by comparing the two values of the exposure *a* and a^*^, $E[Y_{a}]/E[Y_{a^{*}}]$ :

$$\begin{array}{rl} \frac{E[Y_{a}]}{E[Y_{a^{*}}]} & =E_{A\to Y}\times E_{A\to LY}\times E_{A\to M\to Y}\\ & =\frac{E[Y_{aL_{a^{*}}M_{a^{*}}}]}{E[Y_{a^{*}L_{a^{*}}M_{a^{*}}}]}\times \frac{E[Y_{aL_{a}M_{a}}]}{E[Y_{aL_{a^{*}}M_{aL_{a^{*}}}}]}\times \frac{E[Y_{aL_{a^{*}}M_{aL_{a^{*}}}}]}{E[Y_{aL_{a^{*}}M_{a^{*}}}]}, \end{array}$$

where *E_A_*_→_*_Y_*, *E_A_*_→_**_L_***_Y_*, and *E_A_*_→_**_M_**_→Y_ correspond to effects (1), (2), and (3), respectively. However, as shown by Daniel et al,^12^ this decomposition is not unique, and there are six similar decompositions in total (eTable 1). Thus, we used the geometric means of the six estimates as summary measures. In this case, the total effect can also be decomposed into the product of three effects: *E_A_*_→_*_Y_*, *E_A_*_→_**_L_***_Y_*, and *E_A_*_→_**_M_**_→Y_.

Under the identification assumptions described in eMaterial 1, the identification formula for $E[Y_{aL_{a^{*}}M_{a^{**}L_{a^{*}}}}]$ is as follows:

$$\begin{array}{rl} & E[Y_{aL_{a^{*}}M_{a^{**}L_{a^{*}}}}]\\ & \;=\sum_{c}\sum_{l}\sum_{m}E[Y|a,l,m,c]p(m|a^{**},l,c)p(l|a^{*},c)p(c). \end{array}$$

We used a Cox regression model to estimate the expected 10-year incidence of CRC, *E* [*Y* |*a*, **l**, **m**, **c**]. We used Breslow’s estimator to estimate the baseline survival function.^34^ We fit normal linear regression models on the mediators **M** and **L** to estimate *p* (**m** |a^*^, **l**, **c**) and *p* (**l** |a^*^, **c**). We combined the results for all imputed datasets using Rubin’s rule with the number of imputed datasets *K* = 100.^35^ For sensitivity analysis, we performed an analysis with *K* = 10 using the pattern mixture model under the “missing not at random” assumption.^35^ We also conducted sub-site analyses of colon and rectal cancers.

Furthermore, we performed causal mediation analysis as described above in the nested case-control study, which included 513 participants (174 cases and 339 controls) with biomarker data and no missing potential confounding factors using the inverse probability weighting method with design-based sampling probabilities.^36^ Inverse probability weighting analysis of the nested case-control study uses the inverse of design-based sampling probability that each subject is sampled for the analysis during the entire follow-up periods as the weight. Since the sampling probability is known by the nested case-control design, we do not need additional assumptions on the missingness mechanism.^36^^,^^37^ See eMaterial 1 for a detailed description of the statistical analysis.

## RESULTS

Among the 44,271 men eligible to participate in this study at baseline, 32,100 men had a BMI of <25.0 kg/m^2^, 8,542 men had a BMI between 25.0 and 27.4 kg/m^2^, and 3,629 men had a BMI of ≥27.5 kg/m^2^ (Table 1). The plasma concentrations of leptin, C-peptide, and CRP were higher and those of adiponectin were lower among men in the higher BMI categories than among men with a BMI <25.0 kg/m^2^.

**Table 1.** Baseline demographic and clinical characteristics of 44,271 men from the Japan Public Health Center-based study cohort

Characteristics		BMI category, kg/m^2^		

		<25.0	25.0–27.4	≥27.5
Participants, *n*		32,100	8,542	3,629
BMI, kg/m^2^, mean (SD)		22.1 (1.8)	26.0 (0.7)	29.2 (1.8)
Age, years, mean (SD)		51.5 (8.1)	50.8 (7.5)	50.4 (7.3)
Pack-years of smoking, %	Non-smoker	22.4	28.3	30.8
	<20	19.5	18.4	16.3
	<40	37.4	31.2	29.2
	≥40	20.7	22.0	23.8
Alcohol consumption, %	Non-drinker	22.6	21.7	23.0
	Occasional	8.3	10.7	12.7
	<150 g ethanol/week	25.3	23.6	21.2
	<300 g ethanol/week	22.7	22.0	18.8
	<450 g ethanol/week	13.2	13.1	12.4
	≥450 g ethanol/week	8.0	8.8	11.9
Physical activity during leisure time, %	Rarely	65.2	63.0	64.6
	1–3 days/month	16.1	16.7	15.1
	1–2 days/week	9.4	11.1	10.0
	3–4 days/week	4.2	4.6	5.2
	Almost everyday	5.1	4.7	5.1
Family history of cancer, %	No	78.0	78.3	79.0
	Yes	22.0	21.7	21.0
Leptin,^a^ ng/mL		2.8 (2.1–3.7)	4.3 (3.2–5.7)	4.3 (3.3–5.8)
Adiponectin,^a^ µg/mL		4.5 (3.2–6.2)	3.4 (2.5–4.8)	3.3 (2.4–4.7)
C-peptide,^a^ ng/mL		2.3 (1.4–3.7)	2.8 (1.8–4.5)	2.8 (1.8–4.4)
CRP,^a^ mg/L		0.5 (0.3–1.2)	0.9 (0.4–1.9)	0.9 (0.4–2.0)

BMI, body mass index; C-peptide, connecting peptide; CRP, C-reactive protein; SD, standard deviation.

^a^Medians (interquartile ranges) from 100 imputed datasets.

Table 2 illustrates the associations of BMI and plasma concentrations of leptin, adiponectin, C-peptide, and CRP with the risk of CRC. Compared with that for a BMI of less than 25.0 kg/m^2^, the confounder-adjusted hazard ratios (HRs) for a BMI between 25.0 and 27.4 kg/m^2^ and a BMI of ≥27.5 kg/m^2^ were 1.15 (95% confidence interval [CI], 1.00–1.32) and 1.47 (95% CI, 1.22–1.77), respectively. In contrast, upon addition of biomarkers to the model, the HR of BMI was close to 1 (BMI 25.0–27.4 kg/m^2^: HR 0.91; 95% CI, 0.67–1.22; and BMI ≥27.5 kg/m^2^: HR 1.16; 95% CI, 0.84–1.59). Plasma leptin and C-peptide concentrations were associated with an increased risk of CRC (confounder-adjusted HR for a one-unit increment: plasma leptin, 1.59; 95% CI, 1.00–2.54; plasma C-peptide, 1.36; 95% CI, 0.97–1.91). When confounding factors, BMI, and all four biomarkers were added to the model, the association was attenuated, yet persisted with an HR of 1.35 (95% CI, 0.72–2.55) and 1.22 (95% CI, 0.85–1.75) for a one-unit increment of plasma leptin and C-peptide, respectively.

**Table 2.** Adjusted HRs for BMI and biomarkers on the incidence of colorectal cancer based on the data of 44,271 men from the Japan Public Health Center-based study cohort

Variable	Level	Confounding factor-adjusted^a^			Confounding factors + biomarkers-adjusted^b^		

		HR	95% CI	*P*-value	HR	95% CI	*P*-value
BMI, kg/m^2^	<25.0	Reference			Reference		
	25.0–27.4	1.15	1.00–1.32	0.057	0.91	0.67–1.22	0.507
	≥27.5	1.47	1.22–1.77	<0.001	1.16	0.84–1.59	0.374
Leptin^c^	One unit increment	1.59	1.00–2.54	0.051	1.35	0.72–2.55	0.346
Adiponectin^c^	One unit increment	0.72	0.46–1.13	0.148	0.83	0.50–1.37	0.468
C-peptide^c^	One unit increment	1.36	0.97–1.91	0.076	1.22	0.85–1.75	0.287
CRP^c^	One unit increment	1.12	0.93–1.36	0.233	1.06	0.86–1.29	0.598

BMI, body mass index; CI, confidence interval; CRP, C-reactive protein; C-peptide, connecting peptide; HR, hazard ratio; SD, standard deviation.

^a^HRs were adjusted for potential confounding factors, including age, area, pack-years of smoking, alcohol consumption, leisure-time physical activity, and family history of cancer.

^b^HRs were estimated using the model including the above potential confounding factors, BMI, and all four biomarkers.

^c^SDs of log-transformed leptin, adiponectin, C-peptide, and CRP levels (averages of SDs from 100 imputed datasets) were 0.47, 0.51, 0.70, and 1.13, respectively. For the analysis including Biomarker data, the number of imputations = 100.

There were six ways to decompose the total effects of BMI into three path-specific effects (eTable 1). Table 3 and Table 4 show the summary effects for each path with 95% CIs, which are the weighted averages of the direct or indirect effects of BMI in the six decompositions based on multiple imputation (Table 3) and on the inverse probability weighting method with design-based sampling probabilities (Table 4), respectively. By MI analysis, in both BMI categories, there were comparatively large indirect effects that are mediated by circulating leptin and adiponectin (BMI 25.0–27.4 kg/m^2^: risk ratio [RR] 1.29; 95% CI, 0.98–1.69; BMI ≥27.5 kg/m^2^: RR 1.28; 95% CI, 0.98–1.68) and little evidence of direct effects or indirect effects that are not mediated by circulating leptin and adiponectin. Similar results were obtained using the inverse probability weighting method with design-based sampling probabilities: there were large indirect effects through circulating leptin and adiponectin (BMI 25.0–27.4 kg/m^2^: RR 1.26; 95% CI, 1.11–1.40; BMI ≥27.5 kg/m^2^: RR 1.49; 95% CI, 1.06–1.89).

**Table 3.** Summary path-specific effects of BMI category on the incidence of colorectal cancer based on the data of 44,271 men from the Japan Public Health Center-based study cohort

	BMI 25.0–27.4 kg/m^2^		BMI ≥27.5 kg/m^2^	

	Summary RR	95% CI	Summary RR	95% CI
Total effect	1.12	0.95–1.32	1.43	1.18–1.74
Direct effect	0.88	0.65–1.19	1.13	0.82–1.54
Indirect effect 1	1.29	0.98–1.69	1.28	0.98–1.68
Indirect effect 2	0.99	0.92–1.05	0.99	0.93–1.05

BMI, body mass index; CI, confidence interval; RR, risk ratio (reference category of BMI is <25.0 kg/m^2^).

Direct effect: Direct effects of obesity, which are possibly mediated by unmeasured or currently unknown factors. Indirect effect 1: indirect effects of obesity mediated by circulating leptin and adiponectin. Indirect effect 2: indirect effects of obesity that are not mediated by circulating leptin and adiponectin but by hyperinsulinemia and chronic inflammation, represented by circulating C-peptide and C-reactive protein, respectively. The number of imputations = 100.

**Table 4.** Summary path-specific effects of BMI category on the incidence of colorectal cancer based on data from the nested case-control study in the Japan Public Health Center-based study cohort of 513 men

	BMI 25.0–27.4 kg/m^2^		BMI ≥27.5 kg/m^2^	

	Summary RR	95% CI	Summary RR	95% CI
Total effect	1.24	0.79–1.81	1.25	0.47–3.88
Direct effect	0.94	0.63–1.38	1.00	0.40–2.29
Indirect effect 1	1.26	1.11–1.40	1.49	1.06–1.89
Indirect effect 2	1.05	0.81–1.36	0.83	0.65–1.86

BMI, body mass index; CI, confidence interval; RR, risk ratio (reference category of BMI is <25.0 kg/m^2^).

Direct effect: Direct effects of obesity, which are possibly mediated by unmeasured or currently unknown factors. Indirect effect 1: indirect effects of obesity mediated by circulating leptin and adiponectin. Indirect effect 2: indirect effects of obesity that are not mediated by circulating leptin and adiponectin but by hyperinsulinemia and chronic inflammation, represented by circulating C-peptide and C-reactive protein, respectively.

The indirect effects mediated by circulating leptin and adiponectin continued to be present in the sensitivity analyses under the “missing not at random” assumption (eTable 2). The results of the sub-site analyses were similar to the main analysis (eTable 3).

## DISCUSSION

In this study, we decomposed the total causal effects of obesity on the risk of CRC into three path-specific components: (1) direct effects, which are possibly mediated by unmeasured or currently unknown factors; (2) indirect effects that are mediated by circulating leptin and adiponectin; and (3) indirect effects that are not mediated by circulating leptin and adiponectin, but by hyperinsulinemia and chronic inflammation, represented by circulating C-peptide and CRP, respectively. We observed relatively large indirect effects mediated by circulating leptin and adiponectin, which may suggest a major role for these molecules in the pathway connecting obesity and CRC risk.

Few studies have formally examined the mechanistic associations between obesity and CRC risk using mediation analysis.^38^^–^^41^ Ho et al reported that leptin and insulin partially explained the association between waist circumference and the risk of CRC in a Women’s Health Initiative cohort study of postmenopausal women.^38^ Additionally, Aleksandrova et al reported that high-density lipoprotein cholesterol, non-high-molecular-weight adiponectin, and soluble leptin receptors mediate the association between waist circumference and risk of CRC in both men and women according to data from the European Prospective Investigation into Cancer and Nutrition study.^39^ Furthermore, similar results were observed for the association between BMI and CRC in males. Based on the Health Professionals Follow-up Study, Petimar et al recently revealed that metabolic and inflammatory biomarkers jointly mediated the associations of BMI and adult weight gain with the risk of CRC in men, with stronger results for metabolic biomarkers such as adiponectin, C-peptide, and soluble leptin receptors.^41^ Interestingly, leptin and soluble leptin receptors may have been mediators in all three previous studies. These findings are partially consistent with our current findings, even though we were unable to compare our results with those from previous studies due to differences in the number and types of biomarkers under investigation, as well as in the statistical methods used.

In this study, we used a relatively new approach within the causal mediation analysis framework to estimate certain path-specific effects identified from the observed data.^12^^,^^13^ Our main results summarize six different decompositions (Table 3). However, the results from the different decompositions were similar (eTable 1), and all indicated large contributions by pathways involving adipocyte-derived biomarkers. Thus, our conclusion does not depend on the choice of decomposition. Although we were also interested in further decomposing the direct (*A*→**L**→**M**→*Y*) and indirect (*A*→**L**→*Y*) pathways involving adipocyte-derived biomarkers (**L**), we could not identify these effects in the data for methodological reasons.^33^ The decomposition in this study was sufficient to elucidate the importance of these pathways through adipocyte-derived biomarkers.

In the mediation analysis, we created two group variables, **L** and **M**. Table 2 indicates that leptin (*L*_2_) and adiponectin (*L*_1_) had associations with CRC in opposite directions. However, their association with BMI were also in the opposite direction. Therefore, as BMI increased, leptin levels increased leading to an increase in CRC, and as BMI increased, adiponectin levels decreased, also leading to an increase in CRC. This suggests that increased BMI promotes the incidence of CRC through either pathway, indicating that the entire pathway has a positive joint effect. Similarly, as the BMI increased, both CRP (*M*_2_) and C-peptide (*M*_1_) levels increased, leading to an increase in CRC (see Table 1 and Table 2). Therefore, our joint mediators did not pose any significant problems in the interpretation of the results.

A major strength of our study is the use of a relatively new methodology within the causal mediation analysis framework, which allowed us to estimate the effects of mediation underlying several hypothetical pathways based on biological knowledge. However, several limitations of this study should be acknowledged. First, we measured the plasma concentrations of the biomarkers only once per individual. Although some studies have shown moderate to high reproducibility in the assessment of circulating levels of leptin, adiponectin, CRP,^42^ and C-peptide^43^ over a 1-year study period, our results may not be representative of long-term concentrations. Second, inferences from the mediation analyses were partly based on cross-sectional data, although we assumed that, through a series of events, obesity increases biomarkers and subsequently promotes the development of CRC. BMI and biomarkers were assessed cross-sectionally at baseline before cancer diagnosis. Recently, Vansteelandt and Daniel^44^ proposed a different approach for multiple mediators based on the notion of interventional direct and indirect effects. This approach relies on hypothetical randomized interventions on **L** and **M**. Their methodology does not aim to provide the same decomposition as that of the current study but can be used even when the structural dependence between **L** and **M** is unknown. Adaptation of this approach will be important for future research analyses, although it is beyond the scope of this study. Finally, we used MI for the missing biomarker data from the full cohort dataset. In our analysis, the data from the JPHC study included several important predictors of missing biomarker data; however, our main analysis was valid only under the usual “missing at random” assumption. Therefore, we performed a sensitivity analysis using a pattern-mixture model. We found that the results were somewhat robust under the “missing not at random” assumption. Furthermore, the results of the nested case-control study, which used only actual biomarker data and not relied on the missing-at-random assumption under nested case-control sampling, supported the results of the full cohort analyses with imputed biomarker data.

In summary, our mediation analyses revealed that the association between obesity and CRC risk may be largely mediated by a pathway through circulating leptin and adiponectin, suggesting that these adipocyte-derived hormones are fundamental molecules in the pathway connecting obesity and CRC development. Although leptin and adiponectin are closely associated with colorectal carcinogenesis owing to their potential to modify the extent of hyperinsulinemia and chronic inflammation,^2^ some studies have directly related these adipokines to colorectal tumorigenesis via their modulating effects on cell proliferation and survival.^45^^–^^47^ At present, the effects of leptin and adiponectin on the development of CRC have not been fully elucidated and warrant further investigation.

## SUPPLEMENTARY MATERIAL

eMaterial 1. Detailed description of the statistical analysis

eTable 1. All six decompositions of the total effect of BMI are classified as direct or indirect effects

eTable 2. Summary effects (95% CIs) from sensitivity analyses for the violation of the “missing at random” assumption

eTable 3. Summary path-specific effects of BMI category on the incidence of colon and rectal cancers based on data of 44,271 men from the Japan Public Health Center-based Prospective study
