# Supplementary material for: Importance of Circulating Leptin and Adiponectin in the Causal Pathways Between Obesity and the Development of Colorectal Cancer in Japanese Men
Source: J Epidemiol. 2024 Dec 5;34(12):563–9. doi: 10.2188/jea.JE20230148 (PMC11564068; doi:10.2188/jea.JE20230148)
Supplement: Supplementary file 1 [file je-34-563-s001.pdf]

eMaterial 1. Detailed description of the statistical analysis

(A) Assumptions for the identification of the direct and indirect effects

The following four assumptions suffice to identify $E_{A\rightarrow Y}$, $E_{A\rightarrow \mathbf{L}Y}$, and $E_{A\rightarrow \mathbf{M}\rightarrow Y}$ from the observed data: (1) the effect of exposure A on the outcome Y is unconfounded conditional on **C**; (2) the effect of the mediators (**L**, **M**) on the outcome Y is unconfounded conditional on (**C**, A); (3) the effect of exposure A on the mediator (**L**, **M**) is unconfounded conditional on **C**; and (4) none of the mediator-outcome confounding factors are affected by exposure. Stated formally, these four assumptions are (i) $Y_{a\mathbf{lm}} \coprod A | \mathbf{C}$; (ii) $Y_{a\mathbf{lm}} \coprod (\mathbf{L}, \mathbf{M}) | A, \mathbf{C}$; (iii) $(\mathbf{L}_a, \mathbf{M}_a) \coprod A | \mathbf{C}$; (iv) $Y_{a\mathbf{lm}} \coprod (\mathbf{L}_{a*}, \mathbf{M}_{a*}) | \mathbf{C}$.

(B) Method for estimation of direct and indirect effects

For the estimation, we used the following five regression models for the mediators and outcome. We used the Cox regression model to estimate the expected 10-year incidence of CRC, $E\,[Y\,|a$, **l**, **m**, **c**]. We used Breslow's estimator for an estimation of the baseline survival function.[34] We fit four normal linear regression models on the mediators **M** and **L** for the estimation of p (**m** $|a^{*}$, **l**, **c**) and p (**l** $|a^{*}$, **c**), on the basis of factorization p (**m** $|a^{*}*$, **l**, **c**) = p (m_1 $|m_2$, $a^{*}*$, **l**, **c**) p (m_2 $|a^{*}*$, **l**, **c**) and p (**l** $|a^{*}$, **c**) = p (l_1 $|l_2$, a^{*}, **c**) p (l_2 $|a^{*}$, **c**). Using the method of Imai et al.,[14] we performed the following steps to estimate $E[Y_{a\mathbf{L}_{a*}\mathbf{M}_{a**\mathbf{L}_{a*}}}]$. First, for each imputed dataset, the mediators and outcome models were used with the observed data, as described above. Next, $\mathbf{l}^{*}$ and $\mathbf{m}^{*}$ were drawn from the estimated distributions of the mediators $\hat{p}(\mathbf{l}\,|\,a^{*}, \mathbf{c})$ and $\hat{p}(\mathbf{m}\,|\,a^{**}, \mathbf{l}^{*}, \mathbf{c})$. Then, y^{*} was drawn from the estimated distribution of the outcome $\hat{p}(y\,|\,a, \mathbf{l}^{*}, \mathbf{m}^{*}, \mathbf{c})$. Finally, the mean of y^{*} was computed over all individuals.

(C) Method for combining multiple imputed (MI) data

We combined the results for all imputed datasets using Rubin's rule.[35] According to Rubin's rule, combining estimates of the parameter of interest is accomplished by averaging the individual estimates obtained from the analysis of each imputed dataset:

$$\bar{\beta} = \frac{1}{K}\sum_{k=1}^{K}\hat{\beta}_k,$$

for K imputed datasets and point estimates $\hat{\beta}_k$ for the parameter of interest (the logarithms of $E_{A\to Y}$, $E_{A\to \mathbf{L}Y}$, and $E_{A\to \mathbf{M}\to Y}$). Let W_k denote the variance of $\hat{\beta}_k$. The variance estimate of $\bar{\beta}$ was obtained by the following:

$$\mathrm{var}(\bar{\beta}) = \bar{W} + \left(1+\frac{1}{K}\right)B,$$

where $\bar{W} = K^{-1}\sum_{k=1}^{K}W_k$, the average of K imputed variances (the within-imputation component), and $B = (K-1)^{-1}\sum_{k=1}^{K}(\hat{\beta}_k - \bar{\beta})$, the sample variance of point estimates (the between-imputation component). W_k was estimated on the basis of the nonparametric bootstrap for each imputed dataset with 100 resamplings. We set $K = 100$.

(D) Method for the sensitivity analysis

For MI analysis, we used the "missing at random" assumption; in other words, the expected value of the biomarkers was assumed to be the same between the observed and unobserved participants with the same characteristics considered in the imputation model. For sensitivity analysis, we performed an analysis using the pattern mixture model under the "missing not at random" assumption.[35] In particular, we adjusted the imputed values for the participants without blood samples by adding constant values for the natural logarithm of the plasma concentrations of leptin, adiponectin, C-peptide, and CRP as follows:

$$L_j^* = L_j + \delta_{L\,j},\ (j = 1, 2)$$

$M_j^* = M_j + \delta_{Mj}$ (j = 1, 2)

where L_j^* and M_j^* were adjusted values, L_j and M_j were imputed values for the unobserved participants from the regression model, and $\delta_{L\,j}$ and δ_{Mj} were the shift parameters. We set the shift parameters within ± 0.8 SD for each biomarker. For the sensitivity analysis, we set K = 10.

(E) Detailed description of the inverse probability weighing method

Furthermore, we performed the causal mediation analysis as described above in the nested case-control study, which included 513 participants (174 cases and 339 controls) with actual biomarker data and no missing potential confounding factors. According to the design-based sampling probabilities in a nested case-control study that account for additional matching factors of sex, age (within 3 years), date of blood sampling (within 3 months), time since last meal (within 4 hours) and study location (PHC area),[36] the inverse probability weighing method was used to estimate $E[Y_{a\mathbf{L}_{a^*}\mathbf{M}_{a^{**}\mathbf{L}_{a^*}}}]$. Considering a relatively small sample size in the nested case-control study, we repeated the process of generating y^* 1,000 times, and the average of y^* was computed over all individuals and 1,000 simulations. The confidence intervals for the parameter of interest (the logarithms of $E_{A\rightarrow\mathrm{Y}}$, $E_{A\rightarrow\mathbf{L}\mathrm{Y}}$, and $E_{A\rightarrow\mathbf{M}\rightarrow\mathrm{Y}}$) were estimated using the nonparametric bootstrap method with 200 resamplings.

eTable 1. All six decompositions of the total effect of BMI are classified as direct or indirect effects

Here, we define:

$$E_{A\rightarrow Y}(c^{*},c^{**})=\frac{E[Y_{a\mathbf{L}_{c^*}\mathbf{M}_{c^{**}\mathbf{L}_{c^*}}}]}{E[Y_{a^*\mathbf{L}_{c^*}\mathbf{M}_{c^{**}\mathbf{L}_{c^*}}}]},E_{A\rightarrow\mathbf{L}Y}(c,c^{**})\frac{E[Y_{c\mathbf{L}_a\mathbf{M}_{c^{**}\mathbf{L}_a}}]}{E[Y_{c\mathbf{L}_{a^*}\mathbf{M}_{c^{**}\mathbf{L}_{a^*}}}]},E_{A\rightarrow\mathbf{M}\rightarrow Y}(c,c^{*})=\frac{E[Y_{c\mathbf{L}_{c^*}\mathbf{M}_{a\mathbf{L}_{c^*}}}]}{E[Y_{c\mathbf{L}_{c^*}\mathbf{M}_{a^*\mathbf{L}_{c^*}}}]}.$$

For example, the first decomposition for 25.0 kg/m$^2 \leq$ BMI < 27.5 kg/m^2 is as follows:

$$\frac{E[Y_1]}{E[Y_0]}=E_{A\rightarrow Y}(0,0)\times E_{A\rightarrow\mathbf{L}Y}(1,1)\times E_{A\rightarrow\mathbf{M}\rightarrow Y}(1,0)=\frac{E[Y_{1\mathbf{L}_0\mathbf{M}_{0\mathbf{L}_0}}]}{E[Y_{0\mathbf{L}_0\mathbf{M}_{0\mathbf{L}_0}}]}\times\frac{E[Y_{0\mathbf{L}_1\mathbf{M}_{1\mathbf{L}_1}}]}{E[Y_{0\mathbf{L}_0\mathbf{M}_{1\mathbf{L}_0}}]}\times\frac{E[Y_{1\mathbf{L}_0\mathbf{M}_{1\mathbf{L}_0}}]}{E[Y_{1\mathbf{L}_0\mathbf{M}_{0\mathbf{L}_0}}]}.$$

The first component is the contrast of mean outcomes between BMI categories 1 (25.0 ≤ BMI < 27.5) and 0 (BMI< 25) while adiponectin and leptin (**L**), C-peptide and CRP (**M**) were fixed at the levels of BMI category 0 (direct effect). The second component is the effect by changing adiponectin and leptin from the level of BMI category 0 to 1 while the BMI category is fixed at 0 (indirect effect 1). The third component is the effect by changing C-peptide and CRP from the level of BMI category 0 to 1 while the BMI category is fixed at 1 and adipocyte-derived biomarkers fixed at the levels of BMI category 0 (indirect effect 2).

25.0 kg/m$^2 \leq$ BMI < 27.5 kg/m^2

Decomposition	Direct effect	Indirect effect 1	Indirect effect 2
1: $E_{A\rightarrow Y}(0,0)\cdot E_{A\rightarrow\mathbf{L}Y}(1,1)\cdot E_{A\rightarrow\mathbf{M}\rightarrow Y}(1,0)$	0.86	1.32	0.99
2: $E_{A\rightarrow Y}(0,0)\cdot E_{A\rightarrow\mathbf{L}Y}(1,0)\cdot E_{A\rightarrow\mathbf{M}\rightarrow Y}(1,1)$	0.86	1.33	0.98
3: $E_{A\rightarrow Y}(1,0)\cdot E_{A\rightarrow\mathbf{L}Y}(0,0)\cdot E_{A\rightarrow\mathbf{M}\rightarrow Y}(1,1)$	0.92	1.24	0.99
4: $E_{A\rightarrow Y}(0,1)\cdot E_{A\rightarrow\mathbf{L}Y}(1,1)\cdot E_{A\rightarrow\mathbf{M}\rightarrow Y}(0,0)$	0.85	1.33	0.98
5: $E_{A\rightarrow Y}(1,1)\cdot E_{A\rightarrow\mathbf{L}Y}(0,1)\cdot E_{A\rightarrow\mathbf{M}\rightarrow Y}(0,0)$	0.90	1.24	1.01
6: $E_{A\rightarrow Y}(1,1)\cdot E_{A\rightarrow\mathbf{L}Y}(0,0)\cdot E_{A\rightarrow\mathbf{M}\rightarrow Y}(0,1)$	0.90	1.27	0.98

BMI ≥27.5 kg/m^2

Decomposition	Direct effect	Indirect effect 1	Indirect effect 2
1: $E_{A\rightarrow Y}(0,0)\cdot E_{A\rightarrow\mathbf{L}Y}(2,2)\cdot E_{A\rightarrow\mathbf{M}\rightarrow Y}(2,0)$	1.09	1.32	0.99
2: $E_{A\rightarrow Y}(0,0)\cdot E_{A\rightarrow\mathbf{L}Y}(2,0)\cdot E_{A\rightarrow\mathbf{M}\rightarrow Y}(2,2)$	1.09	1.31	0.99
3: $E_{A\rightarrow Y}(2,0)\cdot E_{A\rightarrow\mathbf{L}Y}(0,0)\cdot E_{A\rightarrow\mathbf{M}\rightarrow Y}(2,2)$	1.17	1.24	0.99
4: $E_{A\rightarrow Y}(0,2)\cdot E_{A\rightarrow\mathbf{L}Y}(2,2)\cdot E_{A\rightarrow\mathbf{M}\rightarrow Y}(0,0)$	1.11	1.31	0.98
5: $E_{A\rightarrow Y}(2,2)\cdot E_{A\rightarrow\mathbf{L}Y}(0,2)\cdot E_{A\rightarrow\mathbf{M}\rightarrow Y}(0,0)$	1.14	1.24	1.01
6: $E_{A\rightarrow Y}(2,2)\cdot E_{A\rightarrow\mathbf{L}Y}(0,0)\cdot E_{A\rightarrow\mathbf{M}\rightarrow Y}(0,2)$	1.14	1.27	0.98

Direct effect: direct effects of obesity, which are possibly mediated by unmeasured or currently

unknown factors. Indirect effect 1: indirect effects of obesity that are mediated by circulating leptin and adiponectin. Indirect effect 2: indirect effects of obesity that are not mediated by circulating leptin and adiponectin but by hyperinsulinemia and chronic inflammation, represented by circulating C-peptide and C-reactive protein, respectively.

BMI, body mass index.

The number of imputations = 100.

eTable 2. Summary effects (95% CIs) from sensitivity analyses for the violation of the "missing at random" assumption

	BMI: 25.0–27.4 kg/m^2			BMI: ≥27.5 kg/m^2		
Shift*	Direct effect	Indirect effect 1	Indirect effect 2	Direct effect	Indirect effect 1	Indirect effect 2
+0.2SD	0.78 (0.61–0.98)	1.50 (1.23–1.79)	0.97 (0.90–1.05)	1.00 (0.76–1.28)	1.49 (1.22–1.78)	0.97 (0.90–1.06)
+0.4SD	0.77 (0.65–0.99)	1.51 (1.23–1.72)	0.96 (0.90–1.04)	1.03 (0.80–1.30)	1.45 (1.22–1.71)	0.97 (0.90–1.04)
+0.6SD	0.84 (0.68–1.02)	1.41 (1.20–1.62)	0.96 (0.89–1.03)	1.07 (0.85–1.34)	1.40 (1.19–1.61)	0.96 (0.89–1.03)
+0.8SD	0.88 (0.72–1.06)	1.34 (1.17–1.53)	0.96 (0.89–1.03)	1.12 (0.89–1.39)	1.34 (1.16–1.52)	0.96 (0.89–1.04)
-0.2SD	0.82 (0.64–1.03)	1.43 (1.17–1.71)	0.97 (0.90–1.04)	1.06 (0.80–1.34)	1.41 (1.16–1.70)	0.97 (0.90–1.05)
-0.4SD	0.87 (0.70–1.08)	1.34 (1.12–1.57)	0.96 (0.91–1.02)	1.12 (0.87–1.41)	1.32 (1.12–1.57)	0.97 (0.91–1.02)
-0.6SD	0.93 (0.76–1.13)	1.25 (1.08–1.44)	0.96 (0.92–1.01)	1.19 (0.94–1.47)	1.24 (1.07–1.44)	0.97 (0.91–1.02)
-0.8SD	0.97 (0.80–1.17)	1.19 (1.05–1.35)	0.96 (0.92–1.02)	1.25 (0.99–1.53)	1.18 (1.04–1.34)	0.97 (0.92–1.03)

BMI, body mass index; CI, confidence intervals; SD, standard deviation for each biomarker.
* For adiponectin, the signs of the shift parameters are reversed.

Direct effect: direct effects of obesity, which are possibly mediated by unmeasured or currently unknown factors. Indirect effect 1: indirect effects of obesity that are mediated by circulating leptin and adiponectin. Indirect effect 2: indirect effects of obesity that are not mediated by circulating leptin and adiponectin but by hyperinsulinemia and chronic inflammation, represented by circulating C-peptide and C-reactive protein, respectively.The number of imputations = 10.

eTable 3. Summary path-specific effects of BMI category on the incidence of colon and rectal cancers based on data of 44,271 men from the Japan Public Health Center-based Prospective study

	BMI: 25.0–27.4 kg/m^2		BMI: ≥27.5 kg/m^2	
	Summary RR	95% CI	Summary RR	95% CI
Colon				
Total effect	1.10	0.90–1.34	1.49	1.17–1.90
Direct effect	0.85	0.62–1.18	1.17	0.81–1.68
Indirect effect 1	1.31	0.99–1.71	1.30	0.98–1.71
Indirect effect 2	0.99	0.92–1.06	0.98	0.92–1.05
Rectal				
Total effect	1.18	0.92–1.52	1.31	0.93–1.84
Direct effect	0.90	0.62–1.31	1.00	0.64–1.55
Indirect effect 1	1.34	1.02–1.77	1.34	1.01–1.78
Indirect effect 2	0.98	0.89–1.07	0.97	0.89–1.06

BMI, body mass index; CI, confidence interval; RR, risk ratio (reference category of BMI is <25.0 kg/m^2).

Direct effect: direct effects of obesity, which are possibly mediated by unmeasured or currently unknown factors. Indirect effect 1: indirect effects of obesity that are mediated by circulating leptin and adiponectin. Indirect effect 2: indirect effects of obesity that are not mediated by circulating leptin and adiponectin but by hyperinsulinemia and chronic inflammation, represented by circulating C-peptide and C-reactive protein, respectively. The number of imputations = 100.
